# A computer-aided method for identifying the presence of softwood growth ring boundaries

**DOI:** 10.1371/journal.pone.0235727

**Published:** 2020-09-18

**Authors:** Qizhao Lin, Tuo He, Yongke Sun, Xin He, Jian Qiu

**Affiliations:** 1 College of Material Science and Engineering, Southwest Forestry University, Kunming, Yunnan, China; 2 Research Institute of Wood Industry, Chinese Academy of Forestry, Beijing, China; 3 Wood Collections (WOODPEDIA), Chinese Academy of Forestry, Beijing, China; Wuhan University of Science and Technology, CHINA

## Abstract

The objective of this study was to develop a computer-aided method to quantify the obvious degree of growth ring boundaries of softwood species, based on data analysis with some image processing technologies. For this purpose, a 5× magnified cross-section color micro-image of softwood was cropped into 20 sub-images, and then every image was binarized as a gray image according to an automatic threshold value. After that, the number of black pixels in the gray image was counted row by row and the number of black pixels was binarized to 0 or 100. Finally, a transition band from earlywood to latewood on the sub-image was identified. If everything goes as planned, the growth ring boundaries of the sub-image would be distinct. Otherwise would be indistinct or absent. If more than 50% sub-images are distinct, with the majority voting method, the growth ring boundaries of softwood would be distinct, otherwise would be indistinct or absent. The proposed method has been visualized as a growth-ring-boundary detecting system based on the .NET Framework. A sample of 100 micro-images (see S1 Fig via https://github.com/senly2019/Lin-Qizhao/) of softwood cross-sections were selected for evaluation purposes. In short, this detecting system computes the obvious degree of growth ring boundaries of softwood species by image processing involving image importing, image cropping, image reading, image grayscale, image binarization, data analysis. The results showed that the method used avoided mistakes made by the manual comparison method of identifying the presence of growth ring boundaries, and it has a high accuracy of 98%.

## Introduction

Generally, a wood species can be identified according to the macroscopic and microscopic structural characteristics of the wood, which is a time-consuming process. The traditional methods of wood identification include manual comparison, dichotomous keys, multiple entry keys, punch card search and computer database program search [1]. Researchers have also tried to use DNA molecular marker technology [2], near-infrared spectroscopy technology [3], GC-MS technology [4], computer vision technology [5] and other auxiliary identification methods to improve the accuracy of traditional methods in wood identification and speed the process of identifying wood species.

Tree-ring variables have been shown to be strongly influenced by environmental conditions [6]. The phenomenon of distinct, indistinct or absent growth rings is a wide range of tree-ring research, and its feature is used for wood identification [7]. Recently, researchers have attempted to recognize wood species by utilizing a growth ring boundary detection algorithm [8] such as the Gray Level Co-occurrence Matrix [8, 9], and the color histogram statistical method [10] to extract wood features. Subsequently, various techniques, including Support Vector Machine (SVM) [11], K-nearest neighbor (KNN) ([12–14]), and neural network [15,16], have been used to create many classifiers.

**Fig 1:**
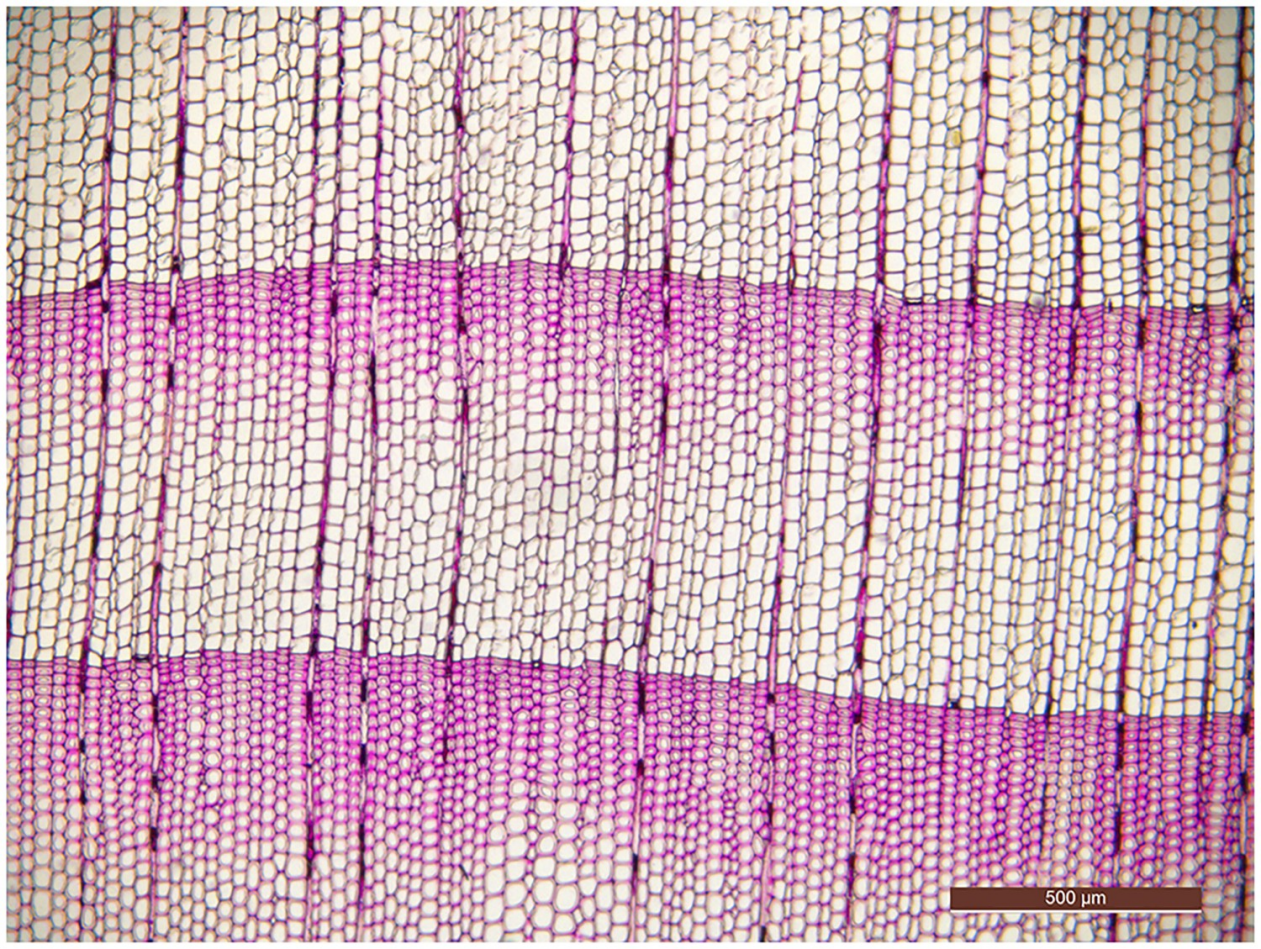
Cross-section of *Tsuga chinensis* var. *forrestii*.

**Fig 2:**
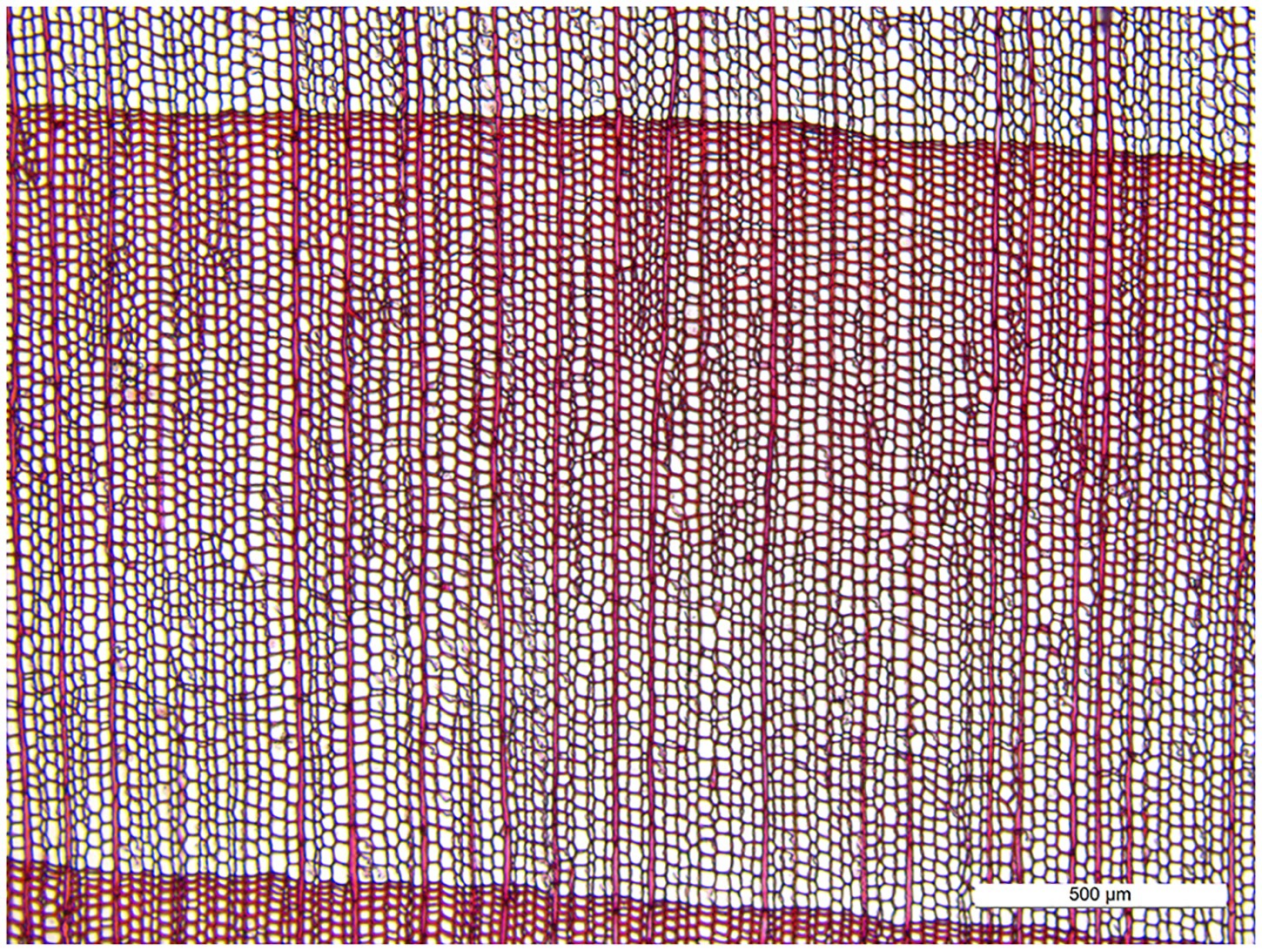
Cross-section of *Podocarpus neriifolius*.

According to the *IAWA list of microscopic features for softwood identification* [17], *Tsuga chinensis* var. *forrestii* (Fig 1) is always identified as having distinct growth ring boundaries, but *Podocarpus neriifolius* (Fig 2) may be recognized as having either obvious growth ring boundaries or not obvious growth ring boundaries [18]. The presence of growth ring boundaries in *Podocarpus neriifolius* varies from person to person, due to definitions of “*growth ring boundaries = growth rings with an abrupt structural change at boundaries between them*” and “*growth ring boundaries indistinct or absent = growth rings boundaries vague and with marked gradual structural changes*” being qualitative, not quantitative, which generates a serious problem for a wood identification researcher.

There is no tool available to quantify the obvious degree of growth ring boundaries in softwood species, although many tools can be used to analyze wood anatomy images, for example, the image analysis tool ROXAS can be used to recognize annual rings in large samples (linear and circular) with > 100 annual rings (see www.wsl.ch/roxas), and to build centuries-long tracheid-lumen chronologies in conifers [19] or quantify plasticity in vessel grouping [20]. DENDRO-2003 densitometer can be used to measure the density profiles of tree ring [21]. Besides, WinDENDRO can measure ring-width manually on sampled cross-sections [22]. They can not judge whether the softwood growth ring boundaries are obvious.

To address this issue, this paper aim to develop a computer-aided method to identify automatically and quantitatively whether there are distinct growth ring boundaries present in softwood species, and provide a powerful quantitative wood anatomy tool [23] making the identification of softwood species more objective and more efficient, in contrast to the method of identifying tree species [8–16].

## Materials and methods

### Image acquisition

A total of 100 microscopic slides were collected from Wood Collections, Chinese Academy of Forestry, representing 100 species (see S1 Table included in https://github.com/senly2019/Lin-Qizhao/), involving 8 families of Ginkgoaceae, Araucariaceae, Podocarpaceae, Cephalotaxaceae, Taxaceae, Pinaceae, Taxodiaceae, and Cupressaceae. These slides were prepared following wood anatomical steps: (i) The cores were cut into small (1cm × 1cm × 2cm) pieces; (ii) Thin sections (ca.15μm) were cut with a microtome; (iii) These sections were stained with safranin and permanently fixed with Gum Arabic. Imaging was performed with a digital camera (LEICA DMC4500) mounted on a light microscope (LEICA DM2000 LED). Images of 2560 × 1920 pixels were captured at 5× magnification using Leica Application Suite (Version 4.9.0). Numerical analysis and data visualization were carried out using Origin8.0.

### Proposed methods

The description of the method includes three parts. 1) The flow chart of the method. 2) The image processing techniques used in this study. 3) How to obtain the final result by some digital techniques, which also include data and statistical analysis. A brief introduction of the workflow is given below. Based on the workflow, a visual computer program has been designed by authors.

#### Part I: Flow chart

**Fig 3:**
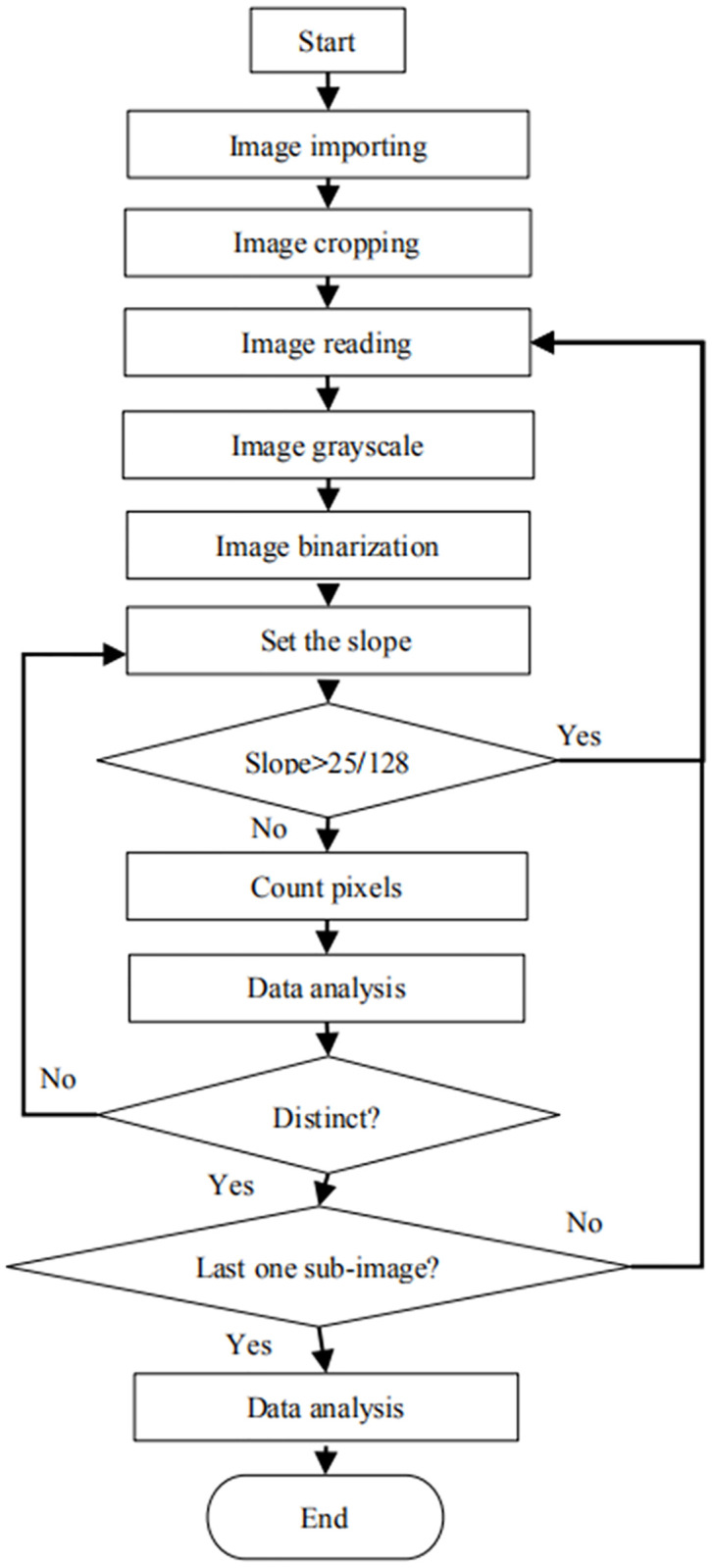
Flow chart of the program.

The flow chart of this method is presented in Fig 3.

The flow chart was described below:

Step 1 Input a microscopy RGB (RGB, R = Red, G = Green, B = Blue) color image collected from cross-section of softwood. The growth rings are parallel to the horizontal direction as far as possible.
Step 2 Crop imported image into 20 sub-images averagely in size along the horizontal direction.
Step 3 Read the images in sequence. If it is successful, then turn to Step 4, otherwise switch to Step 9.
Step 4 Convert a color image into a grayscale image.
Step 5 Calculate a threshold, and then change the grayscale image to get a binary image.
Step 6 Set up the slope value in a loop, if the slope is bigger than the threshold value, turn to Step 3.
Step 7 Count the number of black pixels in each row of the binary image from top to bottom by the slope.
Step 8 Analyze the data generated from Step 7, and then find a row index that meets the specific criteria described below. If it can be found, the growth ring boundaries are distinct and turn to Step 3, otherwise, the growth ring boundaries are indistinct or absent, and turn to Step 6.
Step 9 Statistically analyze all the results output by Step 8. If more than 50% sub-images resulted in distinct growth ring boundaries, then the growth ring boundaries of the sample are distinct, otherwise, they are indistinct or absent.

To operate the program correctly, the detailed instructions and constraints are emphasized as follows.

**Fig 4:**
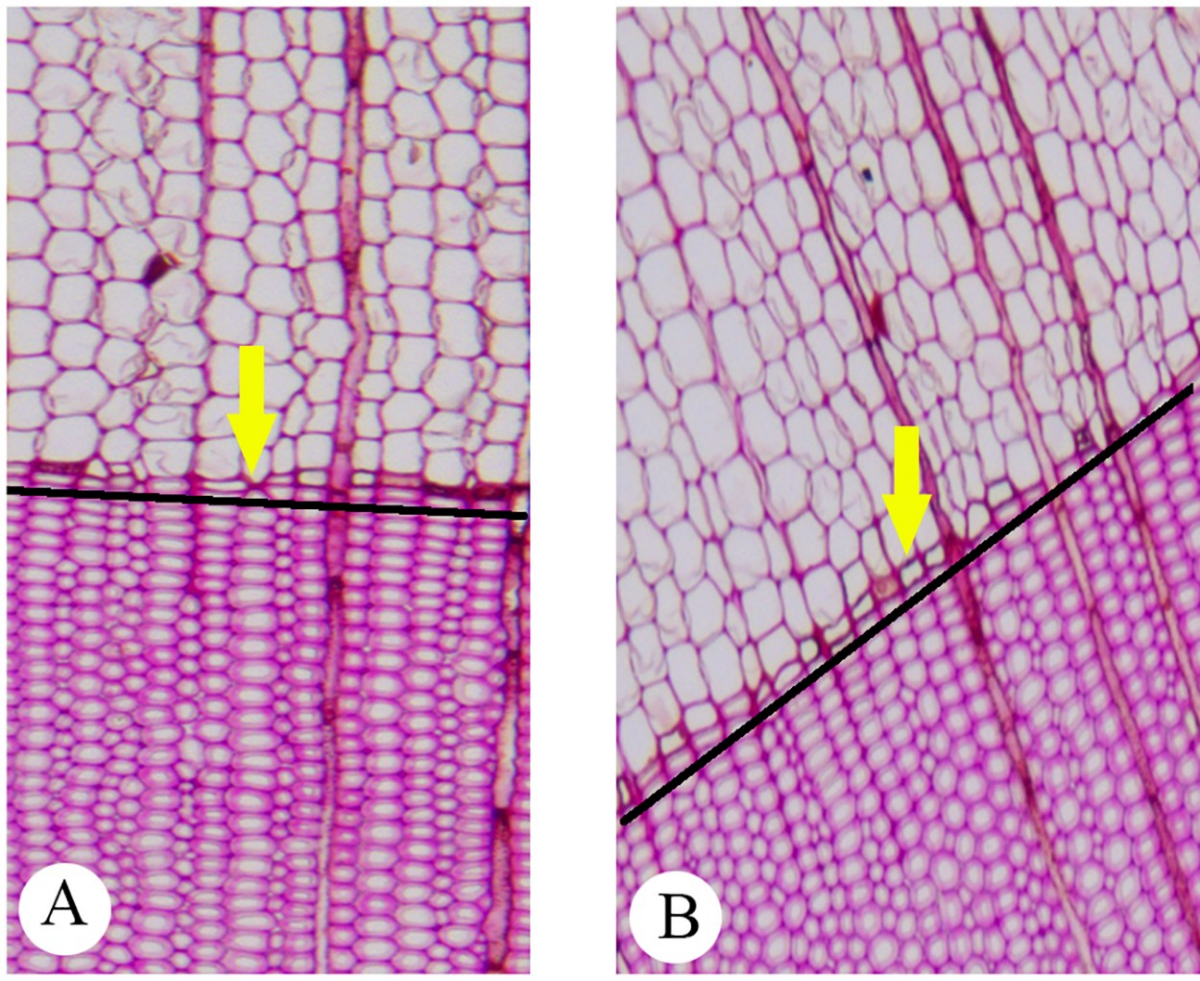
Examples of imported images of local regions: -a: Suitable image. -b: Unsuitable image.

**Fig 5:**
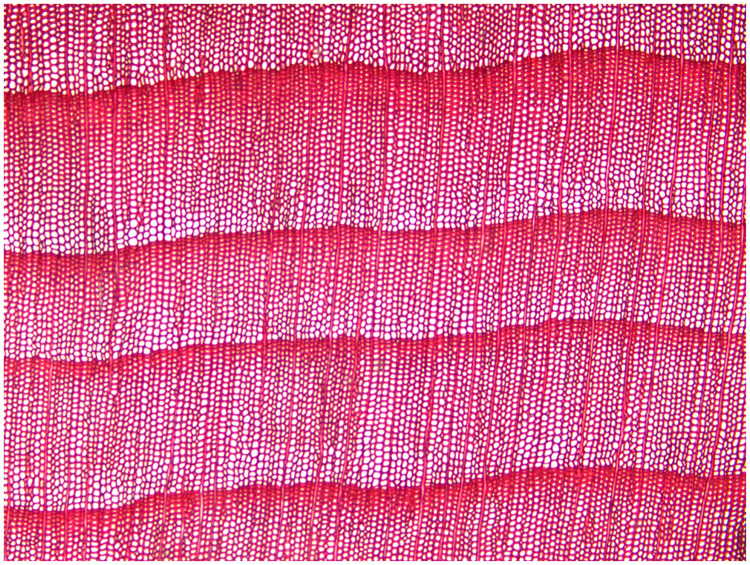
An example of original image for cropping.

**Fig 6:**
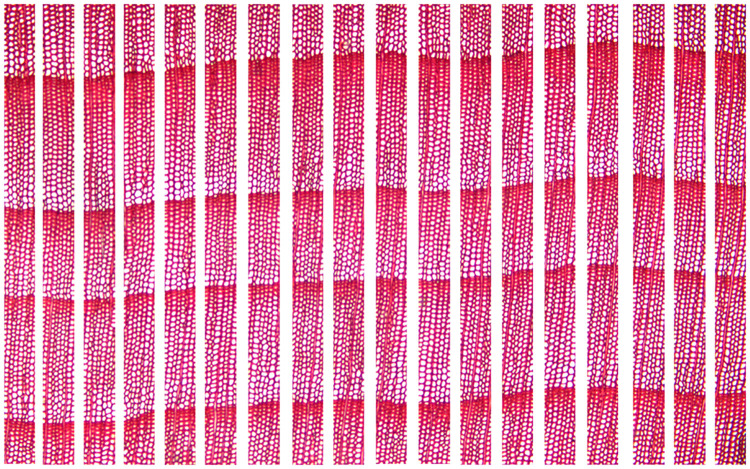
An image set of 20 sub-images after cropping.

1. At Step 1, a suitable image shown as Fig 4A is acceptable but the image like Fig 4B cannot be used, since the slope of the growth ring boundary in Fig 4B is too large. The growth ring on input images should be horizontal. To avoid finding wood rays as the boundary of the growth ring, the maximum acceptable slope of the growth ring boundary designed by the proposed computer-aided method is 0.195 (25 / 128).
2. At Step 2, compared with the original imported image, the sub-image after cropping can reduce the ordinate range of the boundary of the growth ring. Fig 5 shows an original image of a 5× magnified microscopy image of the cross-section of *Taxus wallichiana* without a scale, and the Fig 6 shows an image set of 20 sub-images after image cropping.

#### Part II: Image processing technology

At Step 4, the image processing is performed to find growth rings with an abrupt change at the boundaries. The microscopic RGB images of a cross-section of a softwood species were first stored in a two-dimensional matrix defined as *f(x*,*y)*, converted into a grayscale image, and then changed into a black-and-white binary image. Every pixel point of the color image was calculated by Eq 1 below. The gray value of R, G, B ranges from 0 to 255, and values of all these pixel points lay between 0 to 255 calculated by Eq 1. Image’s dark pixel points represent the tracheid wall thickness, due to tracheid wall is dark but tracheid lumen is light.

Gray=R*0.299+G*0.587+B*0.114 (1)

For segmenting an image, setting up the threshold was simple, efficient, and fast [24]. At Step 5, thresholds were calculated by the program designed by the authors. Threshold values may be changed with different grayscale images. For getting the threshold τ, the program calculated the average value μ and standard deviation σ of all pixel points. These parameters μ, σ, and τ were calculated by Eqs 2, 3, and 4, respectively.

$$\mu =\frac{1}{m\times n}\sum_{j=0}^{n-1}\sum_{i=0}^{m-1}x_{i,j}$$ (2)

$$\sigma =\sqrt{\frac{1}{m\times n}\sum_{j=0}^{n-1}\sum_{i=0}^{m-1}{\left(x_{i,j}-\mu \right)}^{2}}$$ (3)

τ=μ+σ (4)

Where

*x*_*i*,*j*_ is the value of a grayscale image pixel point; *i* is the row index;

*j* is the column index;

*m* is the image height;

*n* is the image width.

The program output various thresholds from different binary images, as shown in Fig 5. By Eq 5, the value of pixel *p(x)* is defined as 0 (black) when if the gray value is less than the threshold τ, otherwise it is defined as 255 (white). After this process, a grayscale image could be converted into a binary image finally.

$$p(x)=\{\begin{array}{cc} 0 & \left(x<\tau \right)\\ 255 & \left(x\geq \tau \right) \end{array}$$ (5)

#### Part III: Mathematical technique

**Fig 7:**
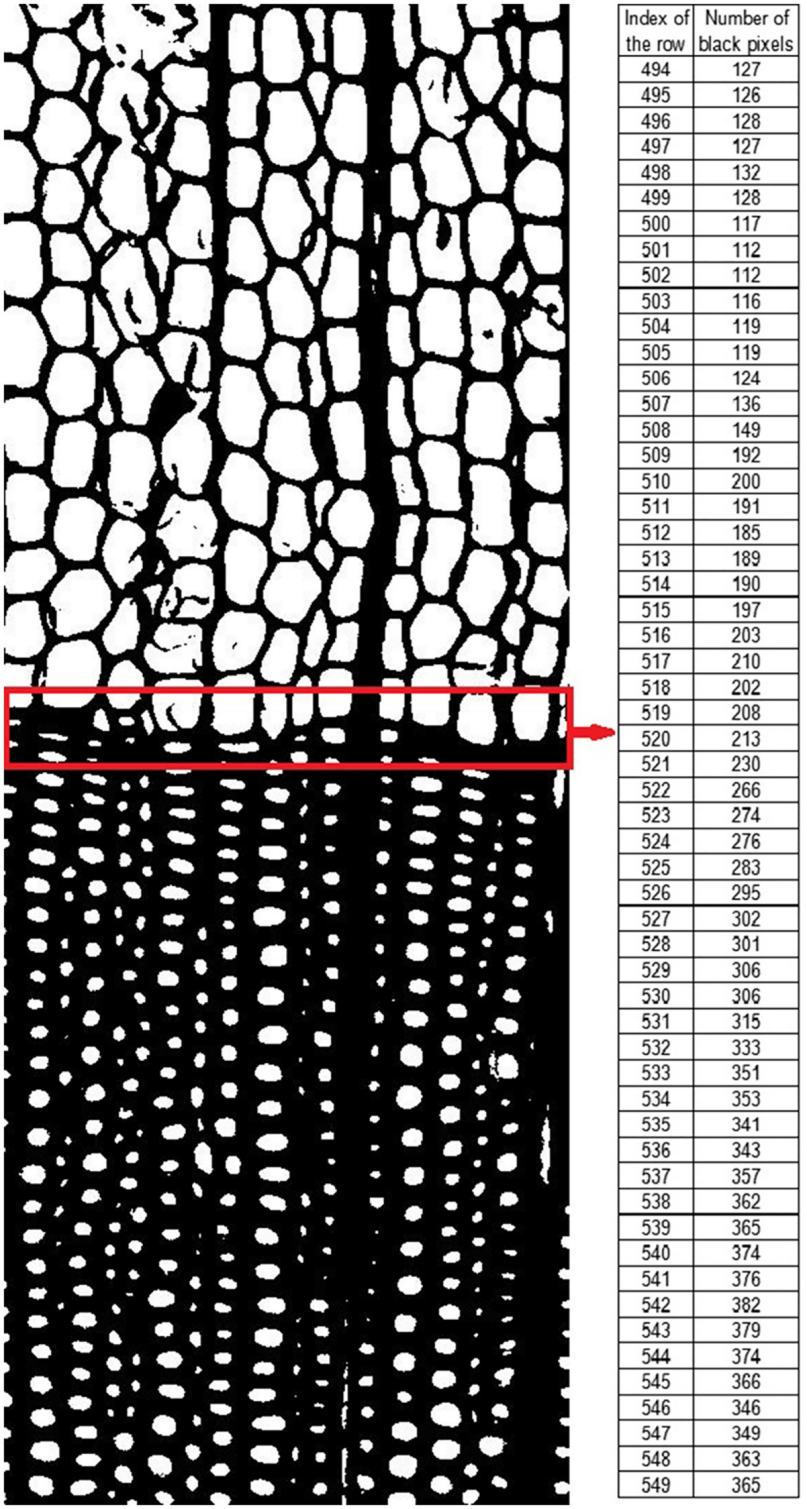
An example of counting black pixels.

A mathematical technique is conducted at Steps 7–9. Fig 7 shows an example of counting black pixels. The first column of the sheet contains row index and the second column contains the counting of black pixels. The first row is at the top of the binary image. The number of black pixels in each row *Y*_*j*_ is counted by Eq 6. In order to find the growth ring boundary, *j* was corrected by Eq 7, where *s* was the slope of the growth ring boundary and computed by Eq 8. The proposed method gets *k* in a loop process. The maximum *j* equaled to *h*-*w*, where *h* is the height of the image.

$$Y_{j}=\sum_{j=0}^{w-1}\frac{p(x_{i,j})}{255}$$ (6)

Where:

*i* is the row index;

*j* is the column index;

and *w* is the width of the image.

$$j\prime =\{\begin{array}{cc} \begin{array}{c} j+s\times x\\ j-s\times w+s\times x \end{array} & \begin{array}{c} s\geq 0\\ s<0 \end{array} \end{array}$$ (7)

$$s=\begin{array}{cc} \frac{k}{w} & \left(1\leq k\leq \frac{w}{10},k\in Z\right) \end{array}$$ (8)

After Step 7, the computer program normalizes these values by Eq 9 at Step 8.

$$YN_{i}=100*\frac{Y_{i}-Y_{\text{min}}}{Y_{\text{max}}-Y_{\text{min}}}$$ (9)

Where:

*YN*_*i*_ is the normalized value of *Y*_*i*_;

*i*, *is* the row index;

*Y*_*min*_ *is* the minimum value of *y*;

*Y*_*max*_ is the maximum value of *y*.

The purpose of normalization operation is to make all scatter plots have the same longitudinal coordinates ranging that from 0 to 100.

The computer program calculates normalized values by Eq 10 and processes normalized values to 0 or 100, which is a binarized operation.

$$YB_{i}=\{\begin{array}{cc} 100 & YN_{i}>=60\\ 0 & YN_{i}<60 \end{array}$$ (10)

Where

*YB*_*i*_ is the binarized value of *YN*_*i*_

*i* is the row index.

**Fig 8:**
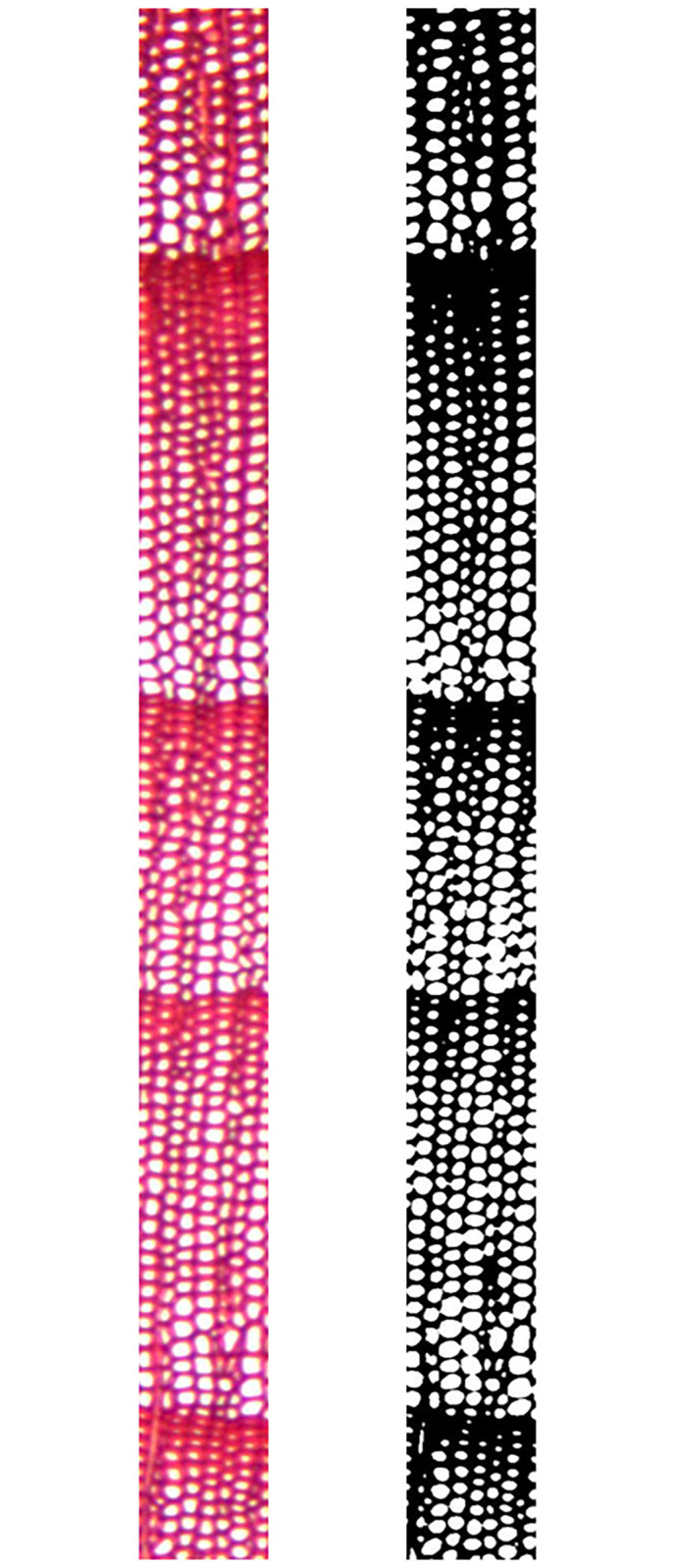
A comparison of sub-images before and after being binarized: -A: Original sub-image; -B: Binarization sub-image.

Fig 8 is a comparison of sum-image before and after being binarized.

**Fig 9:**
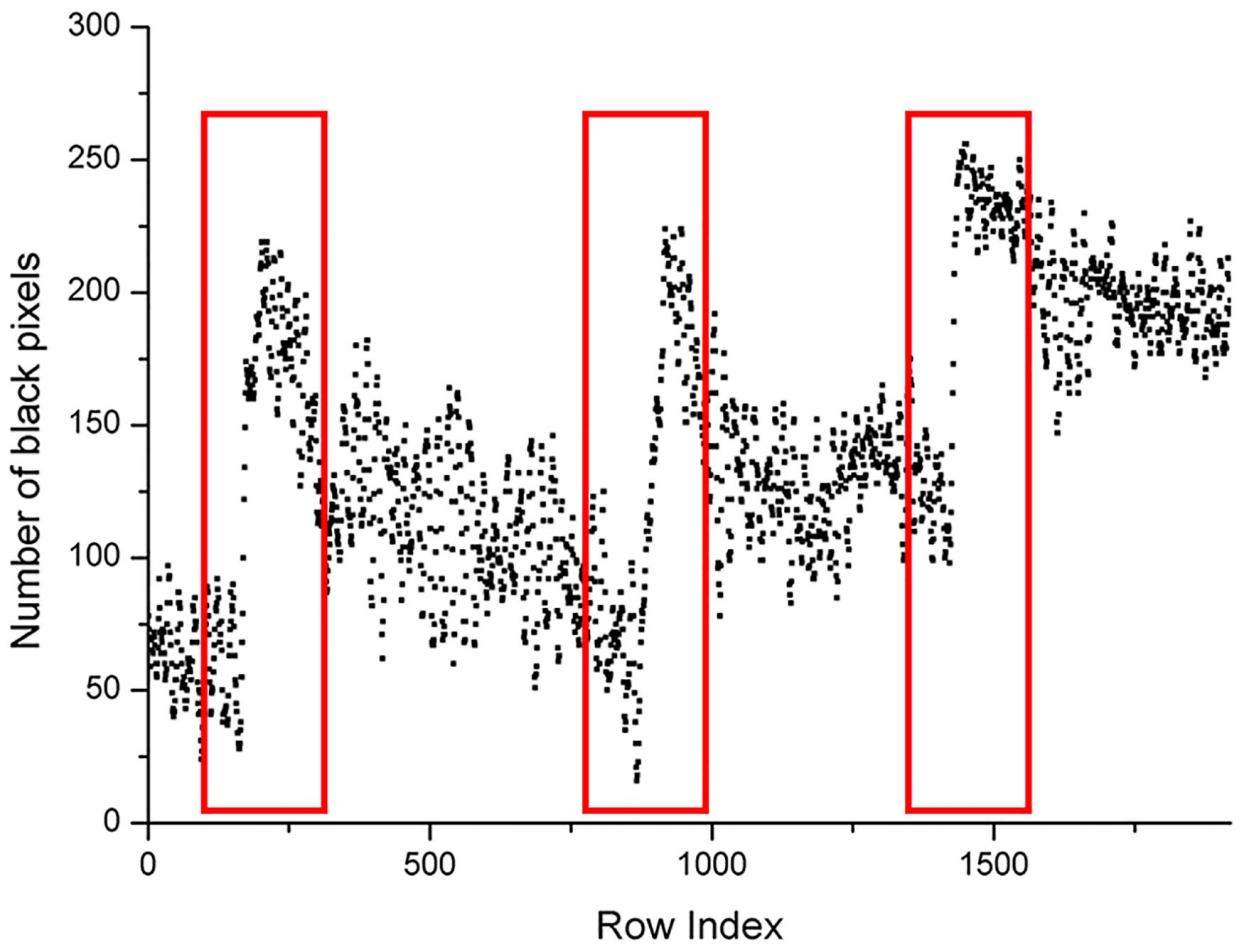
Scatter plot of the number of black pixels of Fig 8B.

**Fig 10:**
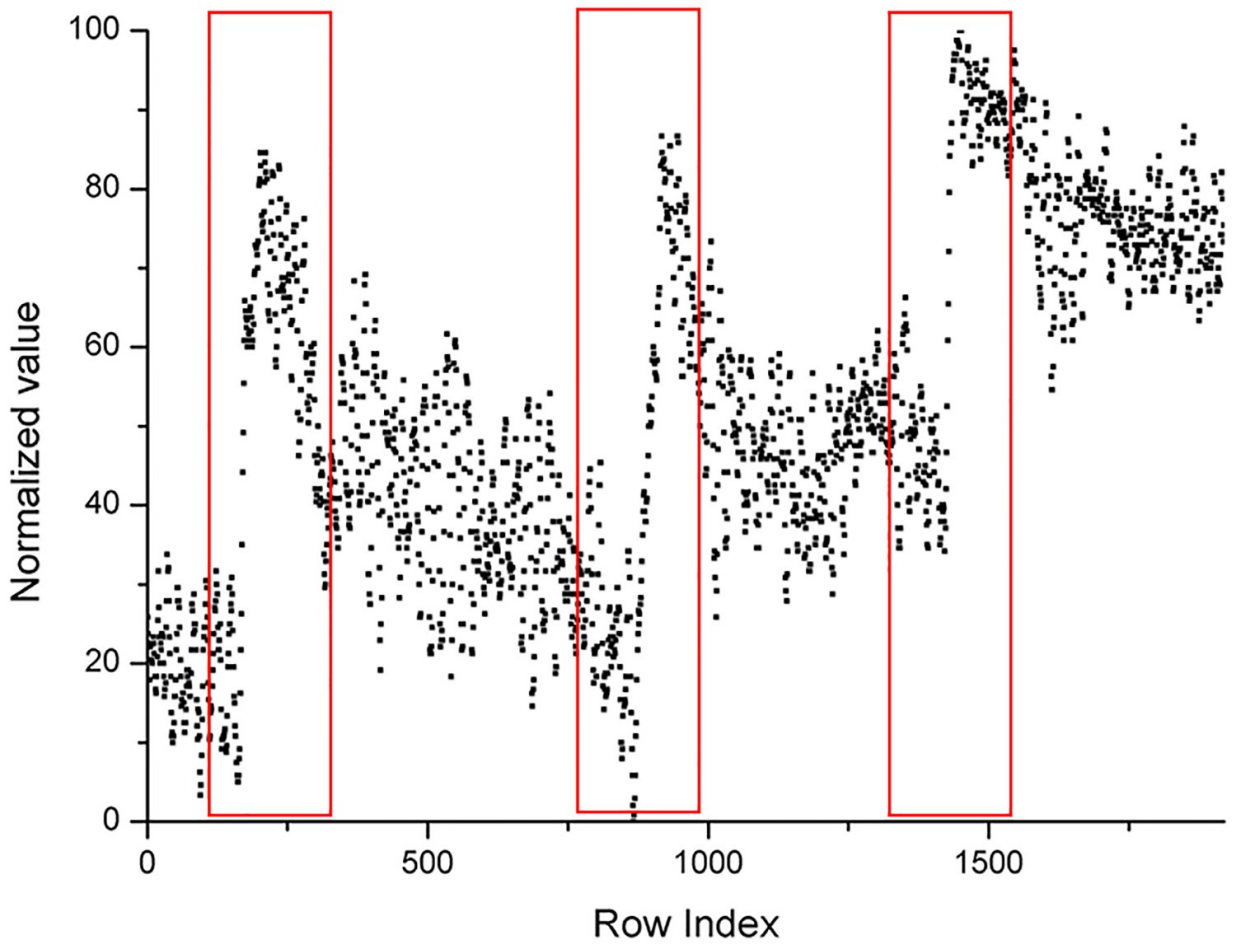
Scatter plot of the number of black pixels of Fig 8B after normalization.

**Fig 11:**
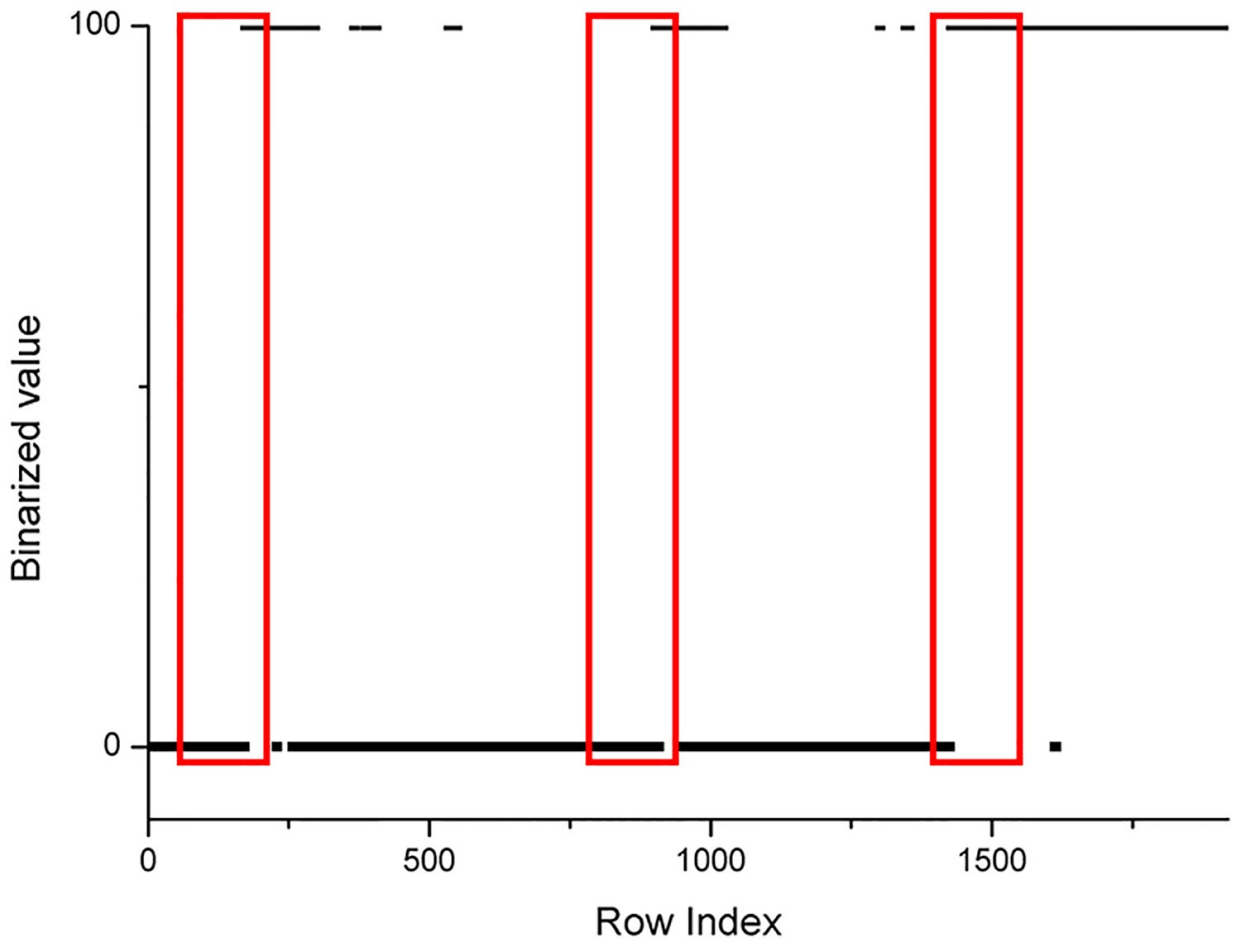
Scatter plot of the number of black pixels of Fig 8B after binarization.

From Figs 9 to 11, it was easy to find out special regions that are labeled by the red rectangular box. These special regions represent growth ring boundaries.

In this study, a sub-method is included at Step 8, which aims to find a row index making *YB*_*i*_ = 0, *YB*_*i+1*_ = 100, $\sum_{i-9}^{i}YB=0$, $\sum_{i+1}^{i+20}YB=100\times 20$, $\frac{\sum_{i+1}^{i+20}y_{i}}{w}>0.3$ and $\frac{\sum_{i+1}^{i+20}y_{i}-\sum_{i-9}^{i}y_{i}}{\sum_{i+1}^{i+20}y_{i}}>0.5$. If it can be found, the growth ring boundaries are defined as “distinct”, otherwise, they are indistinct or absent.

At Step 9, the computer program analyzed the results generated from Step 8. If more than 50% sub-images are reported as “distinct”, then the growth ring boundaries of the sample were distinct, otherwise, they were indistinct or absent.

## Results and discussion

**Fig 12:**
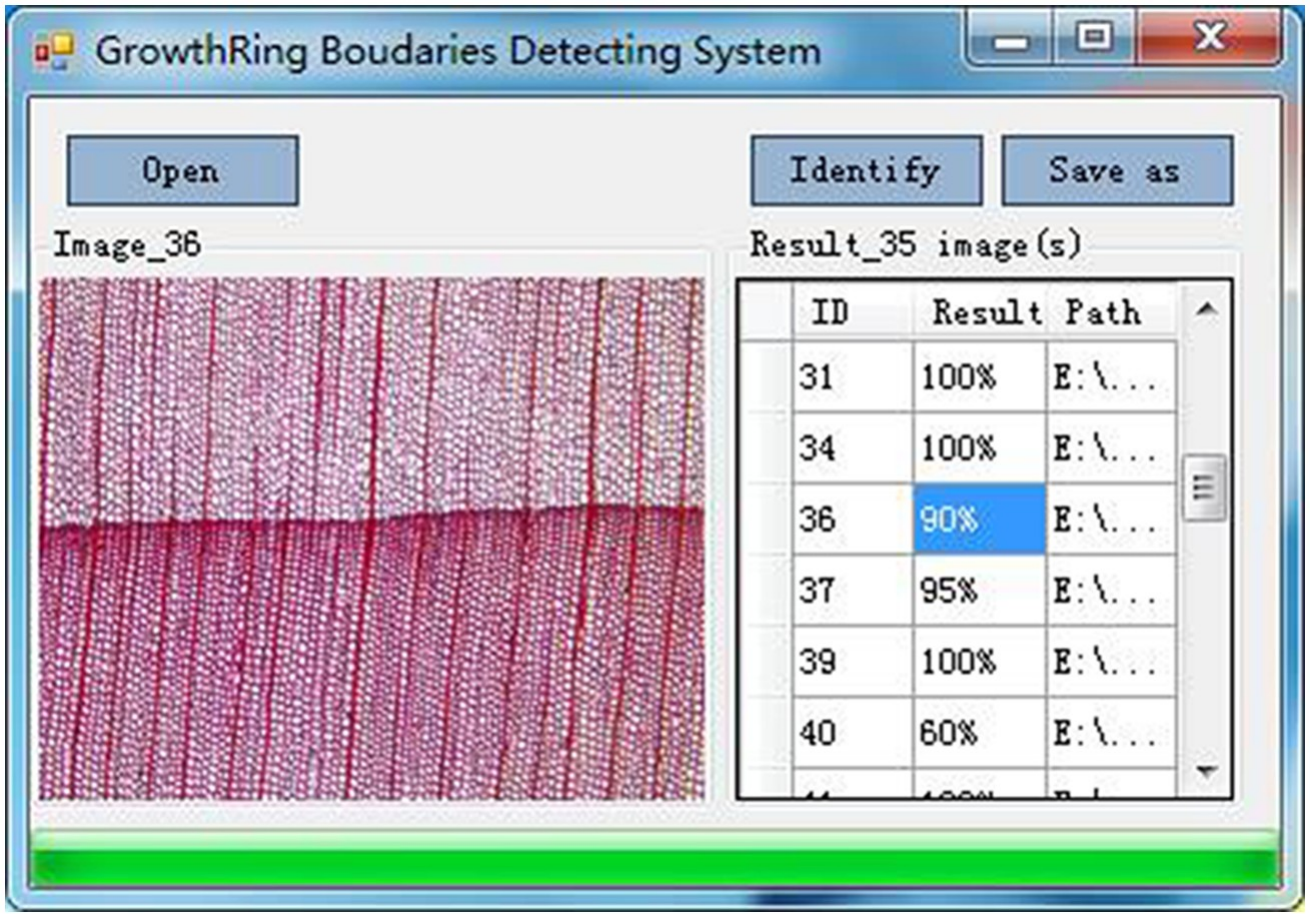
The interface of the program.

For using this method, a visual computer program as shown in Fig 12 was designed with C# based on the .NET Framework. The experimental results are shown in Table 1 including 100 softwood species. All the cross-section micro-images (available via https://github.com/senly2019/Lin-Qizhao/) are 2560×1920 pixels with the same magnification.

**Table 1:** The experimental results of 100 softwood species.

ID	Family	Species	Results of manual method ^a^			Results of the proposed method	
			DC	IC	Qualitative results	Distinct degree (%)	Qualitative results
1	Ginkgoaceae	*Ginkgo biloba*	6	3	Distinct	100	Distinct
2	Araucariaceae	*Araucaria cunninghamii*	0	9	Indistinct or absent	15	Indistinct or absent
3	Podocarpaceae	*Dacrydium pierrei*	**2**	7	**Indistinct or absent**	**80**	**Distinct**
4	Podocarpaceae	*Podocarpus wangii*	9	0	Distinct	100	Distinct
5	Podocarpaceae	*Podocarpus nerii folius*	9	0	Distinct	100	Distinct
6	Cephalotaxaceae	*Cephalotaxus mannii*	4	5	Indistinct or absent	15	Indistinct or absent
7	Cephalotaxaceae	*Cephalotaxus olveri*	9	0	Distinct	100	Distinct
8	Taxaceae	*Amentotaxus argotaenia*	7	2	Distinct	80	Distinct
9	Taxaceae	*Pseudotaxus chienii*	9	0	Distinct	60	Distinct
10	Taxaceae	*Taxus cuspidata*	9	0	Distinct	100	Distinct
11	Taxaceae	*Taxus wallichiana*	9	0	Distinct	100	Distinct
12	Taxaceae	*Taxus wallichiana* var. *mairei*	9	0	Distinct	100	Distinct
13	Taxaceae	*Torreya grandis*	9	0	Distinct	100	Distinct
14	Pinaceae	*Abies beshanzuensis*	9	0	Distinct	100	Distinct
15	Pinaceae	*Abies ernestii*	9	0	Distinct	100	Distinct
16	Pinaceae	*Abies fabri*	9	0	Distinct	100	Distinct
17	Pinaceae	*Abies fargesii*	9	0	Distinct	100	Distinct
18	Pinaceae	*Abies ferreana*	9	0	Distinct	100	Distinct
19	Pinaceae	*Abies forrestii*	9	0	Distinct	100	Distinct
20	Pinaceae	*Abies georgei*	9	0	Distinct	100	Distinct
21	Pinaceae	*Abies georgei* var.*smithii*	9	0	Distinct	100	Distinct
22	Pinaceae	*Abies holophylla*	9	0	Distinct	100	Distinct
23	Pinaceae	*Abies kawakamii*	9	0	Distinct	100	Distinct
24	Pinaceae	*Abies sibirica*	9	0	Distinct	100	Distinct
25	Pinaceae	*Abies squamata*	9	0	Distinct	100	Distinct
26	Pinaceae	*Abies yuanbaoshanensis*	9	0	Distinct	100	Distinct
27	Pinaceae	*Keteleeria davidiana*	9	0	Distinct	100	Distinct
28	Pinaceae	*Keteleeria davidiana* var. *calcarea*	9	0	Distinct	100	Distinct
29	Pinaceae	*Keteleeria evelyniana*	9	0	Distinct	90	Distinct
30	Pinaceae	*Keteleeria fortunei*	9	0	Distinct	100	Distinct
31	Pinaceae	*Keteleeria hainanensis*	9	0	Distinct	95	Distinct
32	Pinaceae	*Keteleeria pubescens*	9	0	Distinct	90	Distinct
33	Pinaceae	*Picea asperata*	9	0	Distinct	85	Distinct
34	Pinaceae	*Picea asperata* var.*aurantiaca*	9	0	Distinct	100	Distinct
35	Pinaceae	*Picea brachytyla*	9	0	Distinct	100	Distinct
36	Pinaceae	*Picea jezoensis* var. *microsperma*	9	0	Distinct	100	Distinct
37	Pinaceae	*Picea koraiensis*	9	0	Distinct	100	Distinct
38	Pinaceae	*Picea purpurea*	9	0	Distinct	100	Distinct
39	Pinaceae	*Pseudotsuga menziesii*	9	0	Distinct	90	Distinct
40	Pinaceae	*Pseudotsuga sinensi*	9	0	Distinct	75	Distinct
41	Pinaceae	*Pseudotsuga sinensis* var. *wilsoniana*	9	0	Distinct	100	Distinct
42	Pinaceae	*Tsuga chinensis*	9	0	Distinct	100	Distinct
43	Pinaceae	*Tsuga chinensis* var. *formosana*	9	0	Distinct	100	Distinct
44	Pinaceae	*Tsuga chinensis* var. *forrestii*	9	0	Distinct	100	Distinct
45	Pinaceae	*Cedrus deodara*	9	0	Distinct	100	Distinct
46	Pinaceae	*Larix griffithiana*	9	0	Distinct	90	Distinct
47	Pinaceae	*Larix mastersiana*	9	0	Distinct	100	Distinct
48	Pinaceae	*Larix olgensis*	9	0	Distinct	100	Distinct
49	Pinaceae	*Larix potaninii*	9	0	Distinct	100	Distinct
50	Pinaceae	*Larix principis-rupprechtii*	9	0	Distinct	100	Distinct
51	Pinaceae	*Larix sibirica*	9	0	Distinct	100	Distinct
52	Pinaceae	*Larix speciosa*	9	0	Distinct	85	Distinct
53	Pinaceae	*Pseudolarix amabilis*	9	0	Distinct	95	Distinct
54	Pinaceae	*Pinus armandi*	9	0	Distinct	100	Distinct
55	Pinaceae	*Pinus bungeana*	9	0	Distinct	100	Distinct
56	Pinaceae	*Pinus fenzeliana*	9	0	Distinct	95	Distinct
57	Pinaceae	*Pinus koraiensi*	9	0	Distinct	100	Distinct
58	Pinaceae	*Pinus parviflora*	9	0	Distinct	100	Distinct
59	Pinaceae	*Pinus densata*	9	0	Distinct	100	Distinct
60	Pinaceae	*Pinus densiflora*	9	0	Distinct	100	Distinct
61	Pinaceae	*Pinus kesiya* var. *langbianensis*	9	0	Distinct	100	Distinct
62	Pinaceae	*Pinus latteri*	9	0	Distinct	95	Distinct
63	Pinaceae	*Pinus massoniana*	9	0	Distinct	100	Distinct
64	Pinaceae	*Pinus nigra*	9	0	Distinct	100	Distinct
65	Pinaceae	*Pinus palustris*	9	0	Distinct	100	Distinct
66	Pinaceae	*Pinus rigida*	9	0	Distinct	90	Distinct
67	Pinaceae	*Pinus roxburghii*	8	1	Distinct	55	Distinct
68	Pinaceae	*Pinus sylvestris*	9	0	Distinct	100	Distinct
69	Pinaceae	*Pinus sylvestris* var. *mongolica*	9	0	Distinct	100	Distinct
70	Pinaceae	*Pinus tabulaeformis*	9	0	Distinct	100	Distinct
71	Pinaceae	*Pinus taeda*	9	0	Distinct	100	Distinct
72	Pinaceae	*Pinus taiwanensis*	9	0	Distinct	100	Distinct
73	Pinaceae	*Pinus thunbergii*	9	0	Distinct	60	Distinct
74	Pinaceae	*Pinus yunnanensis*	9	0	Distinct	100	Distinct
75	Taxodiaceae	*Cryptomeria japonica*	9	0	Distinct	100	Distinct
76	Taxodiaceae	*Cryptomeria japonica* var. *sinensis*	9	0	Distinct	100	Distinct
77	Taxodiaceae	*Cunninghamia lanceolata*	9	0	Distinct	100	Distinct
78	Taxodiaceae	*Glyptostrobus pensilis*	9	0	Distinct	100	Distinct
79	Taxodiaceae	*Taiwania cryptomerioides*	9	0	Distinct	100	Distinct
80	Taxodiaceae	*Taxodium distichum*	9	0	Distinct	85	Distinct
81	Taxodiaceae	*Taxodium distichum* var. *imbricatum*	8	1	Distinct	100	Distinct
82	Taxodiaceae	*Chamaecyparis formosensis*	9	0	Distinct	100	Distinct
83	Taxodiaceae	*Chamaecyparis obtusa* var. *formosana*	5	4	Distinct	90	Distinct
84	Taxodiaceae	*Chamaecyparis pisifera*	9	0	Distinct	100	Distinct
85	Taxodiaceae	*Cupressus duclouxiana*	9	0	Distinct	100	Distinct
86	Cupressaceae	*Cupressus funebris*	9	0	Distinct	100	Distinct
87	Cupressaceae	*Fokienia hodginsii*	9	0	Distinct	100	Distinct
88	Cupressaceae	*Juniperus communis*	9	0	Distinct	100	Distinct
89	Cupressaceae	*Juniperus formosana*	9	0	Distinct	100	Distinct
90	Cupressaceae	*Juniperus rigida*	9	0	Distinct	100	Distinct
91	Cupressaceae	*Sabina chinensis*	9	0	Distinct	100	Distinct
92	Cupressaceae	*Sabina przewalskii*	9	0	Distinct	100	Distinct
93	Cupressaceae	*Sabina recurva*	9	0	Distinct	100	Distinct
94	Cupressaceae	*Sabina squamata*	9	0	Distinct	100	Distinct
95	Cupressaceae	*Sabina tibetica*	9	0	Distinct	100	Distinct
96	Cupressaceae	*Calocedrus macrolepis*	6	3	Distinct	40	Indistinct or absent
97	Cupressaceae	*Calocedrus macrolepis* var. *formosana*	9	0	Distinct	100	Distinct
98	Cupressaceae	*Platycladus orientalis*	9	0	Distinct	100	Distinct
99	Cupressaceae	*Thuja occidentalis*	9	0	Distinct	100	Distinct
100	Cupressaceae	*Thujopsis dolabrata*	9	0	Distinct	100	Distinct

^a^ Manual comparison method; The maximum count is 9. DC means distinct counts, IC means indistinct counts.

As shown in Table 1, the manual comparison method was composed of 9 experts with experience in the identification of softwood to determine whether the boundaries of softwood growth ring were distinct by cross-section micro-image. Among these 100 cross-sections of softwood identified by 9 experts, 91 were identified as an obvious feature of growth ring boundaries by all experts, 1 was identified as a non-obvious feature of growth ring boundaries by all experts.

There were different judgments on 8 cross-sections, as shown in Table 2, different experts have different judgments on the transition type of the growth ring boundaries on these 8 cross-sections of softwood involving 7 families of Ginkgoaceae, Podocarpaceae, Cephalotaxaceae, Taxaceae, Pinaceae, Taxodiaceae, and Cupressaceae. The proposed method provides a quantitative value of the degree of distinctness of growth ring boundaries, and then provides a qualitative conclusion with the majority voting method. Compared with the traditional method [17], to judge whether the growth ring boundaries were distinct, the advantage of the proposed method is providing a qualitative conclusion with the majority voting method based on quantitative computation, which minimized mistakes made by the manual comparison method.

**Table 2:** Different judgments on 8 cross-sections.

ID	Family	Species	Results of the manual method			Results of the proposed method	
			DC	IC	Qualitative results	Distinct degree (%)	Qualitative results
1	Ginkgoaceae	*Ginkgo biloba*	6	3	Distinct	100	Distinct
3	Podocarpaceae	*Dacrydium pierrei*	**2**	**7**	**Indistinct or absent**	**80**	**Distinct**
6	Cephalotaxaceae	*Cephalotaxus mannii*	4	5	Indistinct or absent	15	Indistinct or absent
8	Taxaceae	*Amentotaxus argotaenia*	7	2	Distinct	80	Distinct
67	Pinaceae	*Pinus roxburghii*	8	1	Distinct	55	Distinct
81	Taxodiaceae	*Taxodium distichum* var. *imbricatum*	8	1	Distinct	100	Distinct
83	Taxodiaceae	*Chamaecyparis obtusa* var. *formosana*	5	4	Distinct	90	Distinct
96	Cupressaceae	*Calocedrus macrolepis*	**6**	**3**	**Distinct**	**40**	**Indistinct or absent**

Compared with qualitative results of the manual comparison method which have been run by distinct counts and indistinct counts with the majority voting method [25], qualitative results of the proposed method were different on *Dacrydium pierrei* (*N*_*O*_. 3) of the Podocarpaceae family and *Calocedrus macrolepis* (*N*_*O*_. 96) of the Cupressaceae family after quantifying the distinctness of growth ring boundaries. In other words, the accuracy of the proposed method was 98% assuming that the results of manual comparison were all correct.

**Fig 13:**
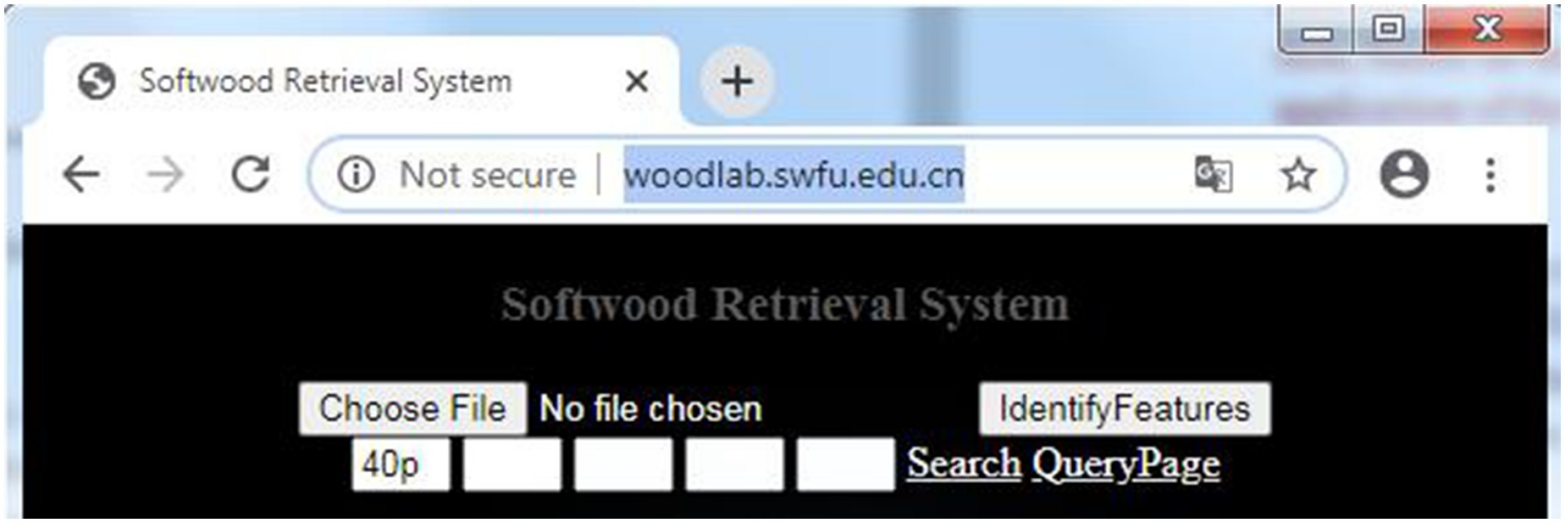
The homepage of softwood retrieval system.

In order to enable more people to use the software to identify whether softwood has distinct growth ring boundaries, the major function has been integrated into the “Softwood Retrieval System”. This website can be accessed at http://woodlab.swfu.edu.cn/, as shown in Fig 13. A cross-section micro-image can been input by clicking “Choose File” button, then feature code of presence of growth ring boundaries will been shown as “40p” or “41p” after “IdentifyFeatures” button clicked, represent distinct and indistinct respectively.

**Fig 14:**
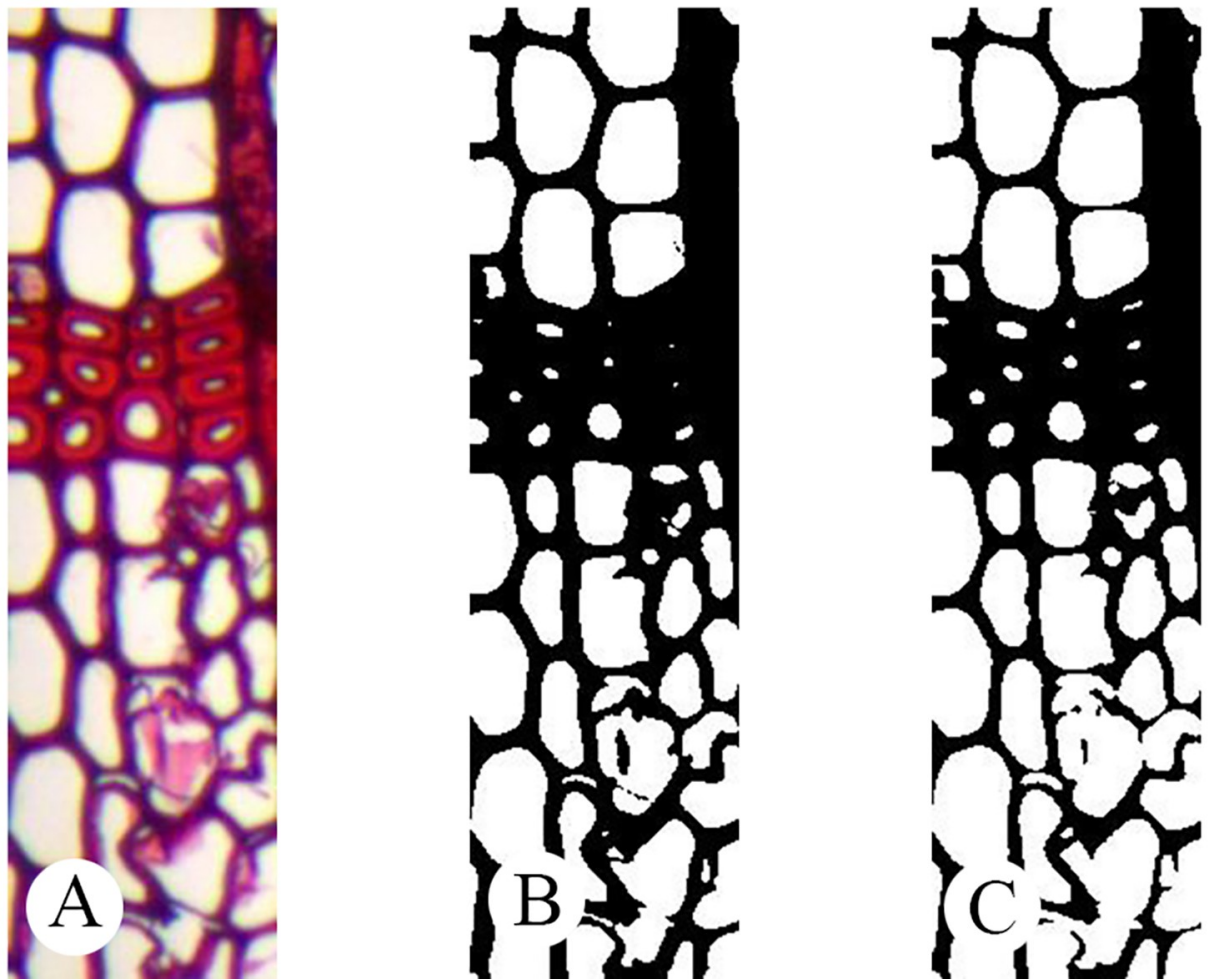
Comparison results of binarization. -A: Original; -B: Processing by an automatic threshold value; -C: Processing by a non-automatic threshold value.

In order to automatically identify whether there are distinct growth ring boundaries present in softwood species, the proposed method used automatic threshold value without human-computer interaction. As shown in Fig 14, the result of automatic threshold binarization is similar to this of non-automatic threshold binarization. Fig 14B and 14C can basically reflect cell wall and cell cavity of Fig 14A.

The proposed method could be improved by combining with a variance which can be used to measure the fluctuation of the image region, to automatically identify the transition from earlywood to latewood of softwood. It could also be applied to assess the distinctiveness of hardwood growth ring boundaries.

## Conclusions

In this paper, a computer-aided method has been proposed for quantifying the obvious degree of growth ring boundaries of softwood species, based on data analysis with some image processing technologies. The proposed method was visualized as a growth-ring-boundary detecting system. A sample of 100 micro-images of softwood cross-sections cut from 100 conifer species were selected for evaluation purposes. In short, this detecting system computes the obvious degree of growth ring boundaries of softwood species by image processing involved image importing, image cropping, image reading, image grayscale, image binarization, data analysis. The results showed that the method has a high accuracy of 98%. In addition, in order to enable more people to use the software to identify whether softwood has distinct growth ring boundaries, the major function has been integrated into the “Softwood Retrieval System”. This website can be accessed at http://woodlab.swfu.edu.cn/. This system can output microscopic feature code as “40p” or “41p” after submitting a cross-section micro-image, represent distinct and indistinct respectively.

Compared with the manual comparison method, our proposed method makes the identification of softwood species more objective and more efficient. The computer-aided method was used instead of the manual operation, which reduced the subjective affection. Automatic methods, such as setting threshold value were employed, which save a lot of time. In the further, we plan to improve performance of “Softwood Retrieval System”. We will collect more samples from various coniferous wood to enhance the generalization performance, and optimize the system in order to convenience the users.

## Supporting information

S1 Fig (TXT)

S1 Table (TXT)
